# Reduced HDL function in children and young adults with type 1 diabetes

**DOI:** 10.1186/s12933-017-0570-2

**Published:** 2017-07-06

**Authors:** Martin Heier, Mark S. Borja, Cathrine Brunborg, Ingebjørg Seljeflot, Hanna Dis Margeirsdottir, Kristian F. Hanssen, Knut Dahl-Jørgensen, Michael N. Oda

**Affiliations:** 1 Children’s Hospital Oakland Research Institute, Oakland, CA USA; 2 Faculty of Medicine, University of Oslo, Oslo, Norway; 3 Oslo Centre for Biostatistics and Epidemiology, Research Support Services, Oslo University Hospital, Oslo, Norway; 4 Center for Clinical Heart Research and Department of Cardiology, Oslo University Hospital, Oslo, Norway; 5 Department of Pediatric and Adolescent Medicine, Akershus University Hospital, Lørenskog, Norway; 6 Department of Endocrinology, Oslo University Hospital, Oslo, Norway; 7 Pediatric Department, Oslo University Hospital, Oslo, Norway

**Keywords:** High-density lipoprotein, Type 1 diabetes, Atherosclerosis, HDL function, HDL-apoA-I exchange

## Abstract

**Background:**

Patients with type 1 diabetes (T1D) are at increased risk of cardiovascular disease (CVD). Measures of high-density lipoprotein (HDL) function provide a better risk estimate for future CVD events than serum levels of HDL cholesterol. The objective of this study was to evaluate HDL function in T1D patients shortly after disease onset compared with healthy control subjects.

**Methods:**

Participants in the atherosclerosis and childhood diabetes study were examined at baseline and after 5 years. At baseline, the cohort included 293 T1D patients with a mean age of 13.7 years and mean HbA1c of 8.4%, along with 111 healthy control subjects. Their HDL function, quantified by HDL-apoA-I exchange (HAE), was assessed at both time points. HAE is a measure of HDL’s dynamic property, specifically its ability to release lipid-poor apolipoprotein A-I (apoA-I), an essential step in reverse cholesterol transport.

**Results:**

The HAE-apoA-I ratio, reflecting the HDL function per concentration unit apoA-I, was significantly lower in the diabetes group both at baseline, 0.33 (SD = 0.06) versus 0.36 (SD = 0.06) %HAE/mg/dL, p < 0.001 and at follow-up, 0.34 (SD = 0.06) versus 0.36 (SD = 0.06)  %HAE/mg/dL, p = 0.003. HAE-apoA-I ratio was significantly and inversely correlated with HbA1c in the diabetes group. Over the 5 years of the study, the mean HAE-apoA-I ratio remained consistent in both groups. Individual changes were less than 15% for half of the study participants.

**Conclusions:**

This study shows reduced HDL function, quantified as HAE-apoA-I ratio, in children and young adults with T1D compared with healthy control subjects. The differences in HDL function appeared shortly after disease onset and persisted over time.

## Background

Patients with type 1 diabetes (T1D) are at an increased risk of morbidity and mortality from cardiovascular disease (CVD) [1, 2]. The underlying mechanisms are only partly understood and even when traditional risk factors for CVD have been addressed, patients with diabetes bear significant residual risk for CVD [3].

In clinical studies high-density lipoprotein cholesterol (HDL-C) levels are consistently inversely associated with coronary heart disease events and mortality [4, 5]. Despite the overall inverse association of HDL-C with CVD risk, nearly 40% of men with coronary heart disease have normal HDL-C levels [6], and very high HDL-C levels are associated with increased risk for major coronary events [7]. Thus, HDL-C levels alone do not provide a complete explanation for the atheroprotective effects of HDL, suggesting that not all HDL are functionally equivalent, and that the cardioprotective nature of HDL is not accurately represented by circulating HDL-C levels. Consequently, the focus has shifted to measures of HDL function, which have yielded a better assessment of CVD risk than HDL-C quantification [8, 9].

The cell-based measure of serum cholesterol efflux capacity (CEC) is the “gold standard” method to assess the efficiency of an individual’s HDL to facilitate reverse cholesterol transport (RCT) [10], a process by which cholesterol is removed from lipid-laden macrophages and delivered primarily to the liver and steroidogenic organs. Clinical studies have demonstrated a strong inverse relationship between CEC and prevalent and incident CVD [11–13]. Recently, a study of 1972 patients in the Dallas Heart Study reported significantly improved atherosclerotic CVD risk assessment when adding CEC to family history, coronary calcium score and C-reactive protein [9]. These studies demonstrate the importance of including CEC in determining cardiovascular risk-prediction indexes. However, since the CEC assay is cell-based, it is difficult to standardize and apply on an individual basis. Furthermore, hypertriglyceridemia has been implicated in yielding artifactually elevated measures of CEC [14]. Combined, these issues complicate the CEC assay’s implementation in a high throughput clinical diagnostic laboratory [8].

**Fig. 1:**
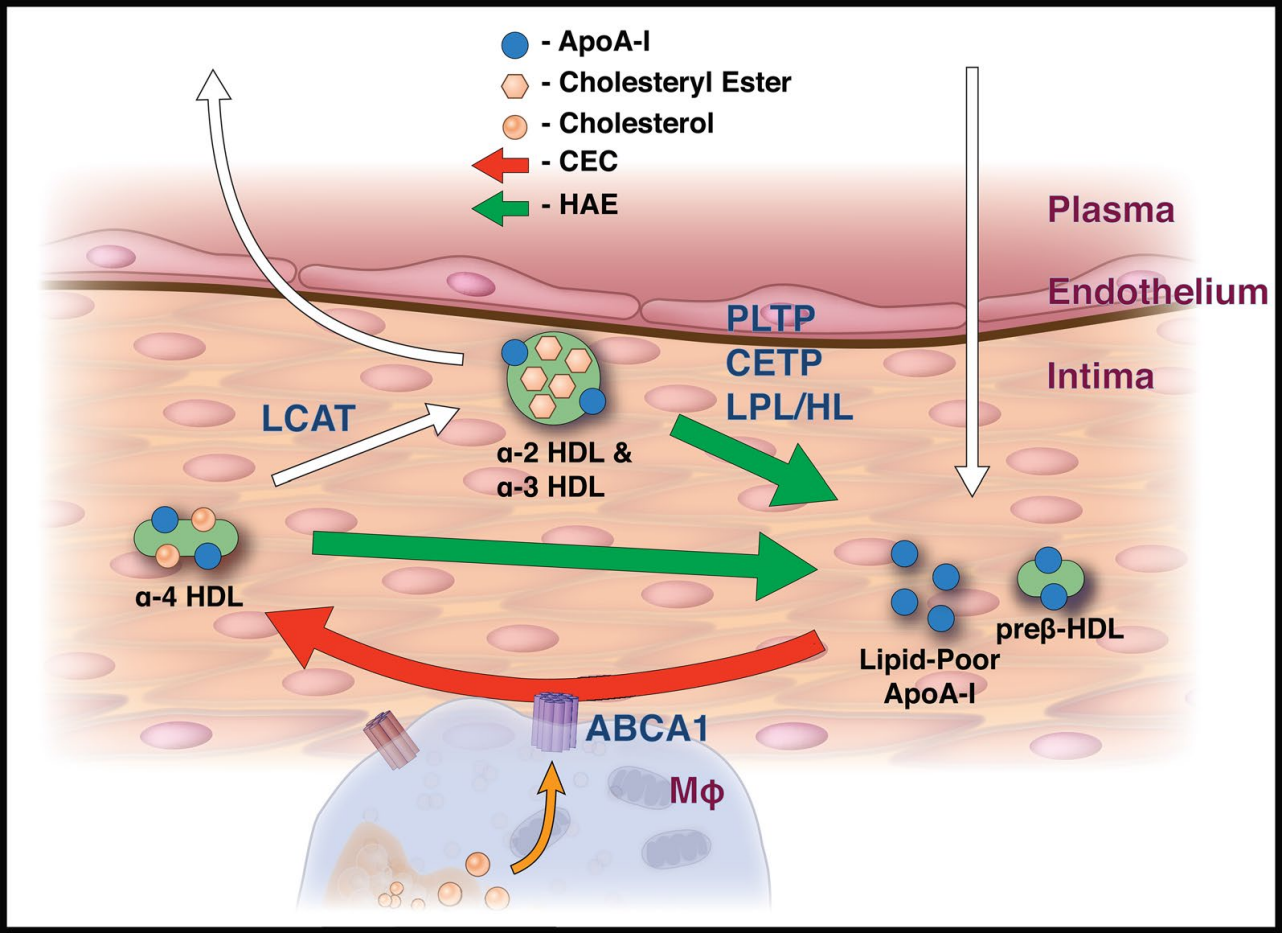
Cholesterol transport in the intima and measurements of HDL function. To facilitate cholesterol efflux from cholesterol-laden macrophages (MΦ), lipid-poor apoA-I binds to ABCA1. During its association with ABCA1, apoA-I acquires free cholesterol (FC) and phospholipid (PL) to form discoidal alpha HDL (*red arrow*). These particles are acted upon by LCAT and converted to cholesterol ester core containing alpha HDL. ApoA-I is liberated from alpha HDL by the action(s) of phospholipid transfer protein (PLTP), cholesterol ester transfer protein (CETP), lipoprotein lipase (LPL) and hepatic lipase (HL) to generate lipid-poor apoA-I and preβ HDL (*green arrows*). The formation of lipid-poor apoA-I and preβ by HDL is a rate-limiting step of this process. These processes can be quantified by the CEC (*red arrow*) and HAE (*green arrows*) assays

Apolipoprotein A-I (apoA-I), the main protein component of HDL, is an important mediator of RCT by promoting lipid efflux via membrane-bound ATP-binding cassette transporter A1 (ABCA1) [15, 16]. The ability of HDL to release lipid-poor apoA-I, the primary substrate for de novo HDL biogenesis via ABCA1, has been hypothesized as the rate-limiting step of RCT (Fig. 1) [17]. ApoA-I undergoes significant conformational change upon association/dissociation with HDL, and this transition can be quantified by electron paramagnetic resonance (EPR) or fluorescent methods as a measure of HDL-apoA-I exchange (HAE) [18–20]. HAE is a discriminating measure of HDL function, distinguishing healthy from CVD states in both animals and humans [18]. Furthermore, HAE strongly correlates with the CEC assay (r = 0.69, p < 0.001) in subjects with normolipidemic profiles [21]. HAE calculated per unit HDL, as the HAE-apoA-I ratio, yields a measure of HDL function analogous to enzymatic specific activity and is more discriminating of CVD status than HAE alone [21].

We hypothesized that residual CVD risk in diabetes patients is at least in part derived from early onset and sustained HDL dysfunction in these individuals. To test this hypothesis, we quantified HDL function by the HAE assay in children and young adults with T1D.

## Methods

### Study population

The atherosclerosis and childhood diabetes (ACD) study started in 2006, and included 314 children and adolescents with T1D along with 120 healthy control subjects. They were 8–18 years old and resided in the South East health region of Norway. The inclusion process and details of the cohort have been described previously [22, 23]. From 2011 to 2013, a 5-year follow-up was conducted, and 80% of the original participants were examined along with 38 newly recruited control subjects. The HAE assay was performed on serum samples from 293 patients and 111 control subjects at baseline and 241 patients and 128 control subjects at follow-up.

Written informed consent was directly provided by participants over 18 years of age and by at least one parent for participants less than 18 years of age. The protocols at baseline and follow-up have both been approved by the Norwegian Regional Committee for Medical and Health Research Ethics. The study was conducted according to the Declaration of Helsinki.

### Clinical and laboratory methods

All blood samples were drawn in the morning between 08:00 and 10:00 am after an overnight fast, centrifuged within 1 h at 2500×*g* for 10 min and stored at −80 °C until analysis.

ApoA-I levels were determined by immunoturbidimetry (Roche Diagnostics, Basel, Switzerland), and the coefficient of variation (CV) was 4%. C-reactive protein (CRP) was measured by high sensitivity ELISA (DRG Instruments GmbH, Germany) with a detection limit of 0.1 mg/L. The inter-assay CV was <5%. HbA1c was analyzed at a DCCT-standardized laboratory by high performance liquid chromatography (Variant; Bio-Rad, Richmond, CA, USA), CV <3%. Other routine laboratory analyses were performed by conventional methods.

### HAE assay

Freshly thawed plasma was treated with polyethylene glycol to precipitate apoB-containing lipoproteins and mixed with 3 mg/mL nitroxide spin-labeled apoA-I probe [18]. EPR was performed using a Bruker eScan EPR spectrometer (Bruker BioSpin GmbH, Karlsruhe, Germany) outfitted with a temperature controller (Noxygen Science Transfer & Diagnostics GmbH, Elzach, Germany). Samples were incubated for 15 min at 37 °C and scanned at 37 °C. The peak amplitude of the nitroxide signal from HAE probe in the sample (3462–3470 Gauss) was compared to the peak amplitude of a proprietary internal standard (3507–3515 Gauss) provided by Bruker. The internal standard is contained within the eScan spectrometer cavity and does not contact the sample. Since the y-axis of an EPR spectrum is measured in arbitrary units, measuring the sample against a fixed internal standard facilitates normalization of sample response. HAE activity represents the sample: internal standard signal ratio at 37 °C. The maximal %HAE activity was calculated by comparing HAE activity to a standard curve ranging in the degree of probe lipid-associated signal. All samples were read in duplicate and averaged. The final result reflects the relative efficiency of apoA-I exchange. Intra-assay CV was 4%.

### Carotid intima-media thickness

At baseline, carotid intima-media thickness (cIMT) of the common carotid arteries was acquired using a Siemens Acuson Sequoia 512 ultrasound scanner (Siemens Acuson; Mountain View, CA, USA) equipped with a linear array 14 MHz transducer. Further details of the examination have been described previously [22]. At follow-up, cIMT was determined using a Zonare Z-one Ultra ultrasound scanner (Zonare Medical Systems; Mountain View, CA, USA) equipped with a linear array 8 MHz transducer and analyzed using M’Ath 3.2.0. software (Intelligence in Medical Imaging; Paris, France). All cIMT measurements were from the far wall of the common carotid arteries in end-diastole. When calculating Young’s modulus, blood pressure values recorded by a standard oscillometric device over the brachial artery were used.

### Statistical analysis

Demographic and clinical data are presented as either means with standard deviations (SD), medians with 25th and 75th percentiles or proportions. Student’s t test, or Mann–Whitney U test in case of non-normally distributed data, was applied to test differences in continuous variables between groups. Pearson’s correlation coefficient (r), or Spearman’s rho (ρ) in case of non-normally distributed data, was applied for correlation analyses between continuous variables. Univariate regression analysis was performed to identify associations between HAE-apoA-I ratio as an exposure variable and surrogate markers of atherosclerosis as outcome variables, as well as associations between HAE-apoA-I ratio, gender and HbA1c. Multivariate regression analysis with a backward elimination procedure was performed to determine and control for confounding variables. For model inclusion, these variables were required to be associated with both the exposure and outcome variables with p < 0.05. Multivariate regression analysis with a backward elimination procedure was also applied to identify risk factors for HAE-apoA-I ratio. Variables with a p value <0.20 from univariate analysis were considered as candidates for the multivariate model. In all analyses, statistical significance was assumed for p < 0.05. The software IBM SPSS Statistics for Macintosh, version 19.0 (Armonk, NY: IBM Corp.) was used for all statistical analyses.

## Results

The clinical and metabolic characteristics of the diabetes patients and the healthy control subjects are presented in Table 1. The diabetes patients had higher diastolic blood pressure, HbA1c, total cholesterol, LDL cholesterol, apoB, apoA-I, body weight, body mass index (BMI) and waist circumference compared with controls both at baseline and at the 5-year follow-up. At baseline, when compared with the Norwegian Childhood Diabetes Registry, the cohort was a representative sample of the young T1D population in Norway regarding HbA1c, blood pressure, lipid status as well as gender and stage of puberty [22]. Since patients under 8 years of age were not included, the subjects of this study had diagnosed diabetes for a longer period, had higher BMI, were more frequently insulin pump users and were slightly older than the total population of youth with T1D in Norway.

**Table 1:** Clinical and metabolic characteristics at baseline and follow-up

	Baseline			5-year follow-up		
	Diabetes	Controls	p value	Diabetes	Controls	p value
n	293	111		241	128	
Diabetes duration (years)	5.6 (3.4)			10.3 (3.6)		
Insulin pump users (%)	52.9			61.3		
Age (years)	13.7 (2.8)	13.3 (2.5)	0.175	18.7 (2.8)	18.7 (2.9)	0.895
Girls, n (%)	147 (50.2)	63 (56.8)	0.265	129 (53.5)	72 (56.3)	0.661
Height (cm)	160.4 (14.5)	157.6 (13.3)	0.072	171.2 (9.1)	172.1 (9.2)	0.383
Weight (kg)	54.8 (16.8)	48.4 (13.2)	<0.001	71.0 (14.6)	66.9 (12.5)	0.006
BMI (kg/m^2^)	20.8 (4.0)	19.1 (3.1)	<0.001	24.1 (4.3)	22.5 (3.4)	<0.001
Waist circumference (cm)	71.2 (10.0)	66.8 (6.6)	<0.001	79.0 (9.6)	75.5 (8.2)	< 0.001
Systolic blood pressure (mmHg)	101 (10)	98 (10)	0.018	112 (11)	111 (10)	0.656
Diastolic blood pressure (mmHg)	60 (8)	58 (7)	0.018	70 (8)	68 (8)	0.024
HbA1c (%) (mmol/mol, SD)	8.4 (1.3) [68, 13]	5.3 (0.3) [34, 3]	<0.001	9.0 (1.4) [75, 15]	5.2 (0.3) [3, 33]	<0.001
Total Cholesterol (mg/dL)	178 (31)	166 (27)	0.001	186 (39)	170 (39)	0.001
HDL (mg/dL)	70 (15)	66 (15)	0.065	62 (19)	62 (15)	0.161
LDL (mg/dL)	97 (27)	89 (27)	0.016	104 (31)	97 (31)	0.014
Triglycerides (mg/dL)^a^	62 (44, 80)	62 (44, 80)	0.457	80 (53, 115)	71 (53, 89)	0.024
Apolipoprotein B (mg/dL)	74 (19)	66 (17)	<0.001	90 (26)	79 (23)	<0.001
Apolipoprotein A-I (mg/dL)	154 (28)	147 (27)	0.026	156 (31)	148 (27)	0.006
Urine albumin/creatinine (mg/mmol)^a^	6.2 (3.5, 11.5)	5.7 (3.3, 12.0)	0.637	5.8 (2.7, 13.7)	3.0 (1.7, 8.0)	0.001
HDL-apoA-1 exchange (%)	50.7 (9.8)	52.4 (9.2)	0.105	52.1 (10.4)	52.1 (10.2)	0.960
HAE-apoA-1 ratio (%/mg/dL)	0.33 (0.06)	0.36 (0.06)	<0.001	0.34 (0.06)	0.36 (0.06)	0.003

Mean values (SD)

*HAE* HDL-apoA-I exchange

^a^Median (25th and 75th percentile)

**Fig. 2:**
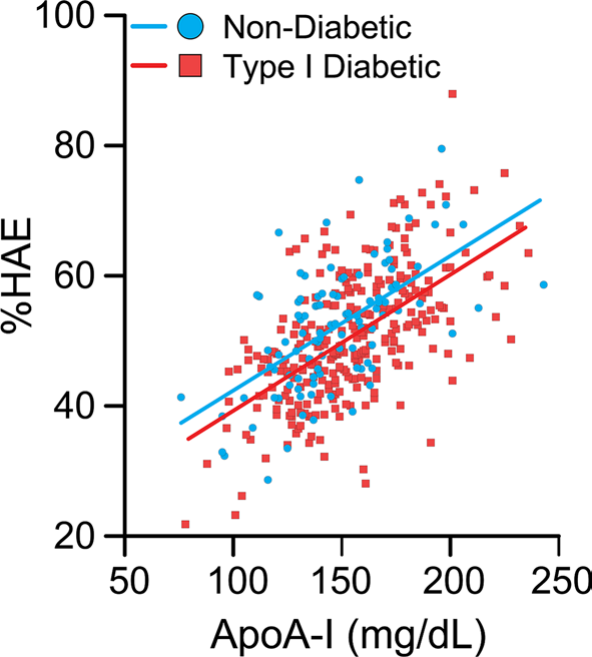
The relationship between  %HAE and apoA-I at baseline. N = 293 T1D patients. N = 111 for healthy control subjects. All resided in the South East region of Norway and had a mean age of 13.7 years. The average  %HAE for the T1D subjects was 50.7 and 52.4 for controls. T1D subjects differed significantly from healthy controls in %HAE-apoA-I (p < 0.001)

The HAE-apoA-I ratio, a measure of HDL function per concentration unit of apoA-I (g/L), was significantly lower in the diabetes group compared with the controls both at baseline and at follow-up (Table 1). The relationship between HAE and apoA-I at baseline is illustrated in Fig. 2.

**Fig. 3:**
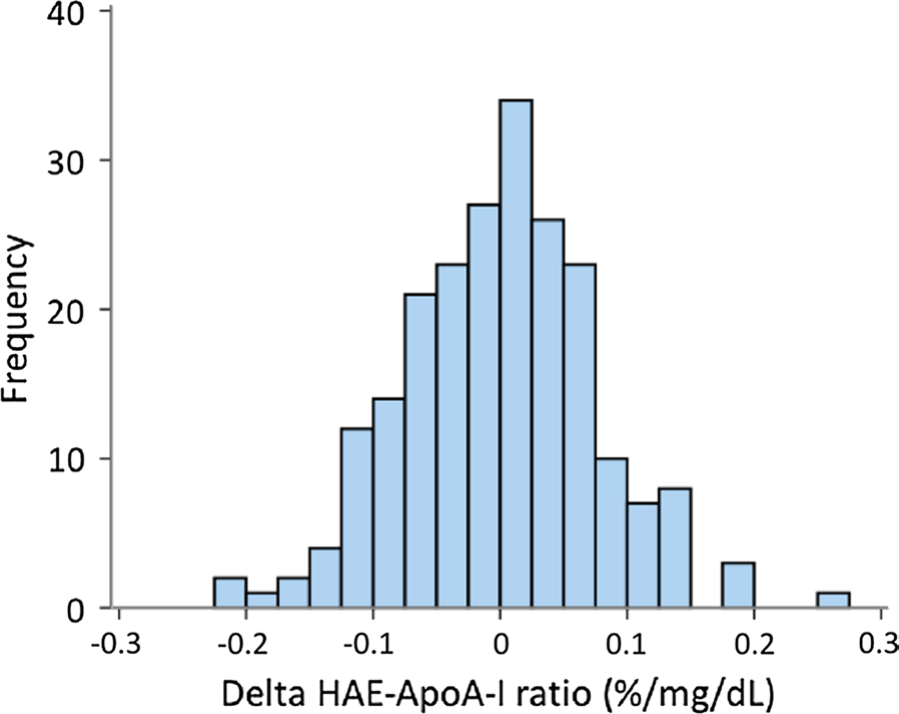
Frequency distribution for delta HAE-apoA-1 ratio in diabetes patients. The delta value is calculated by subtracting the baseline value from the follow-up value of HAE-apoA-I ratio for each participant. N = 218. The values for most patients are close to zero, indicating little change in HAE-apoA-I ratio over the 5 years of the study

The mean HAE-apoA-I ratio for the entire cohort was stable over the 5 years studied (Table 1). In order to evaluate change over time on an individual level, delta variables were created. Values for the delta variables were concentrated around zero, as exemplified by the frequency distribution for delta HAE-apoA-I ratio among diabetes patients (Fig. 3). Furthermore, the standard deviation was 0.08%/mg/dL and the interquartile range was 0.10%/mg/dL in both groups, demonstrating that the individual change in HAE-apoA-I ratio over 5 years was less than 15% for half of the participants.

At baseline, there was no significant gender difference in HAE-apoA-I ratio in either the diabetes group or in the control group. At the 5-year follow-up, however, females had significantly lower HAE-apoA-I ratio than males both in the diabetes group, 0.35 (SD = 0.06) versus 0.33 (SD = 0.05) %/mg/dL, p = 0.041, and in the control group, 0.37 (SD = 0.06) versus 0.35 (SD = 0.05) %/mg/dL, p = 0.047. The association between female gender and HAE-apoA-I ratio remained significant in a linear regression model controlling for pubertal stage. In the diabetes group at the 5-year follow-up, females taking oral contraceptives had significantly lower HAE-apoA-I ratio than females not taking oral contraceptives, 0.34 (SD = 0.05) versus 0.32 (SD = 0.05) %/mg/dL, p = 0.019. There was no difference in the control group. Diabetic females using oral contraceptives also had significantly lower HAE-apoA-I ratio than diabetic males, 0.35 (SD = 0.06) versus 0.32 (SD = 0.05) %/mg/dL, p = 0.002. There was no significant difference between diabetic females not taking oral contraceptives and diabetic males. Among males at the 5-year follow-up, the diabetes patients had significantly lower HAE-apoA-I ratio than control subjects, 0.37 (SD = 0.06) versus 0.35 (SD = 0.06) %/mg/dL, p = 0.029.

The HAE-apoA-I ratio was significantly and inversely correlated with HbA1c, except in the control group at baseline, as shown in Table 2.

**Table 2:** Correlations between HAE-apoA-I ratio and HbA1c at baseline and follow-up

	Baseline		5-year follow-up	
	Pearson’s r	p value	Pearson’s r	p value
Diabetes group	−0.209	<0.001	−0.146	0.025
Control group	−0.044	0.662	−0.279	0.002

In linear multiple regression analyses (Table 3), the association between HAE-apoA-I ratio and HbA1c in the diabetes group at baseline remained significant, B = −0.707, p = 0.007 and R^2^ = 0.094, controlling for total cholesterol levels. In the diabetes group at follow-up, the association was confounded by smoking and total cholesterol levels. In the control group at follow-up, the association remained significant, B = −5.508, p = 0.002 and R^2^ = 0.078, no confounding variables.

**Table 3:** Linear multiple regression analyses

Patient selection	Beta-value	R^2^	Standard error	p value	Variables controlled for
Diabetes group at baseline	−0.707	0.094	0.262	0.007	Total cholesterol
Control group at follow-up	−5.508	0.078	1.703	0.002	None

Significant associations between HAE-apoA-I ratio as the outcome variable and HbA1c as the explanatory variable

Predictive linear regression models to determine risk factors for HDL function were generated for each group (Table 4). In the diabetes group at baseline, insulin pump use, total cholesterol, HDL cholesterol and HbA1c were significant, R^2^ = 0.151 for the model. At follow-up, smoking, HbA1c and HDL cholesterol were significant, R^2^ = 0.108. In the control group the significant risk factors were smoking, and HDL cholesterol at baseline, R^2^ = 0.104, and HDL cholesterol and HbA1c at follow-up, R^2^ = 0.176.

**Table 4:** Predictive linear regression models with HAE-apoA-I ratio as the outcome variable

Patient selection	R^2^ for the model	Significant risk factors	Beta-value	Standard error	p value
Diabetes group at baseline	0.151	Insulin pump use	−1.933	0.673	0.004
		Total cholesterol	−1.244	0.426	0.004
		HDL cholesterol	−2.479	0.823	0.003
		HbA1c	−0.923	0.285	0.001
Diabetes group at follow-up	0.108	HDL cholesterol	−3.031	0.764	<0.001
		Smoking	−5.282	1.970	0.008
		HbA1c	−0.502	0.259	0.054
Control group at baseline	0.104	HDL cholesterol	−4.144	1.377	0.003
		Smoking	−11.715	5.827	0.047
Control group at follow-up	0.176	HDL cholesterol	−4.499	1.202	<0.001
		HbA1c	−4.768	1.655	0.005

There were no significant associations between HDL function and either cIMT, Young’s modulus or CRP at either time point.

## Discussion

In this study, HDL function, as measured by HAE-apoA-I ratio, was reduced in patients with childhood onset T1D in comparison with healthy control subjects. The difference was observed in patients with a mean age of 13.7 years at baseline, and was still present at a follow-up examination 5 years later. This is the first study to examine HAE in a cohort of youth with T1D. Importantly, we demonstrate for the first time that changes in HAE are a sustained effect that occur early in the onset of T1D. HAE is a key aspect of RCT, consequently, these results indicate that reduction in HDL function may be a mechanism underlying the increased risk of CVD seen in T1D. Loss of HAE may be related to HDL’s antioxidant function, which is also impaired by diabetes [24]. The HDL of diabetes patients are unable to reverse the inhibitory effect of oxidized LDL on endothelium-dependent vasorelaxation [25]. As a result, reductions in HAE-apoA-I ratio may be due to the diabetes-associated enhanced inflammation.

Interestingly, there was little change in HAE during the 5 years of follow-up for either diabetes patients or control subjects, both at the cohort and individual level. Delta values showed that for half of the cohort, the HDL function changed less than 15% over 5 years, with increases as common as decreases. Despite a lack of other supporting longitudinal studies, our data indicate that the effect of T1D on HDL function is rapid and persistent.

There was no gender difference in HDL function at baseline. At the 5-year follow-up, however, females had lower HDL function than males, both in the diabetes group and the control group. This difference was not explained by pubertal stage. We have previously demonstrated gender differences in CRP levels in the ACD cohort, and these were largely accounted for by the presence of T1D and use of oral contraceptives [26]. The results of the present study indicate that HDL function is influenced by oral contraceptives in a similar manner, as there was no difference in HDL function between males and females without oral contraceptives. A large study of German adolescents has shown a correlation between oral contraceptives and an unfavorable CVD risk factor profile [27]. They found increased levels of both CRP and HDL in users of oral contraceptives, which is in line with a recent study of adult women [28]. HDL has both pro- and anti-inflammatory capabilities [29], and our results indicate that oral contraceptives may influence this property of HDL. Consequently, reduced HDL function may contribute to the increased risk of CVD seen in females using oral contraceptives [30].

In previous studies of patients with T1D no association between HDL function and HbA1c were demonstrated, possibly due to small sample sizes [24, 25]. Similarly, correlations between HbA1c and HAE in the diabetes patients of this study were quite modest, and the association did not remain significant at follow-up when controlling for confounding variables. The significant association in the control group at follow-up is not easily explained, but might be clinically relevant. In a previous study of subjects without diabetes, elevated levels of HbA1c, though still within normal range, were significantly associated with increased mortality risk [31]. Whether this increased risk is related to impaired HDL function requires further study.

CEC is inversely associated with cIMT and CVD events [11, 12]. Interestingly, despite the well-established correlation between HAE and CEC, in the present study, there were no significant associations between HAE and our surrogate markers of atherosclerosis. This is probably due to the participants’ young age, the relatively short time since the onset of T1D, and the limited presence of atherosclerosis in this age group. The previous studies demonstrating the relationship between CEC and CVD were composed of subjects (30–70 years of age) who had considerably more time to develop CVD than the subjects of the current study [11–13].

The HAE-apoA-I ratio is a measure of HDL function per particle, analogous to measures of enzyme specific activity. The diabetes group exhibited elevated apoA-I levels. Interestingly, diabetes patients and controls exhibit similar HAE. This is likely due to the increase in apoA-I expression in T1D subjects, but whether this increase in apoA-I levels is a regulated compensatory mechanism is presently unknown. Elevated apoA-I in the diabetes group is probably not due to an anomalous selection in our cohort, as other studies show comparable differences [32, 33]. In the present study, HDL function per particle distinguished disease from healthy states better than the total level of HDL function, consistent with prior studies [18].

Inherent strengths of this study include the relatively large number of participants, the longitudinal design and the representative sample of diabetes patients as determined by comparison with the Norwegian Childhood Diabetes Registry. An innovative aspect of the study is the use of the HAE assay to quantify HDL function. The HAE assay requires only 50 µl of plasma, serum or whole blood and doesn’t require isolation of HDL. Instead, it directly assesses HDL function after a 15-min incubation and 80-s read time in the EPR instrument. The simplicity and minimal sample handling of the HAE assay contribute to its high precision (4% coefficient of variability) and day-to-day consistency (5% variability). The limitations of this study include the relatively narrow normal range of HAE response. The 75th percentile is approximately 30% greater than the 25th percentile. Abnormal and normal values may thus be difficult to distinguish. This result may be due to the relatively uniform genetic, social, age and lifestyle characteristics of the cohort. Another limitation is the lack of information about physical fitness and exercise in the cohort. Furthermore, BMI and waist circumference were significantly higher in the diabetes group. Although these measures of body composition were not significant predictors of HDL function in our regression models, neither in the current nor in previous studies, the group disparity may have undetected influence on the results [21].

## Conclusions

This study shows reduced HDL function, as measured by HAE-apoA-I ratio, in children and young adults with T1D compared with healthy control subjects. This difference was detectable early after the onset of T1D and persisted over the 5 years of the study, supporting the hypothesis that HDL function is compromised early and remains a chronic aspect of T1D. A persistent deficit in HDL function is likely a significant contributor to the enhanced CVD risk in these patients.

## Abbreviations

ABCA1: ATP-binding cassette transporter A1
ACD: atherosclerosis and childhood diabetes
ApoA-I: apolipoprotein A-I
BMI: body mass index
CEC: cholesterol efflux capacity
cIMT: carotid intima-media thickness
CRP: C-reactive protein
CV: coefficient of variation
CVD: Cardiovascular disease
EPR: electron paramagnetic resonance
HAE: HDL-apoA-I exchange
HDL: high-density lipoprotein
HDL-C: high-density lipoprotein cholesterol
r: Pearson’s correlation coefficient
ρ: Spearmans’s rho
RCT: reverse cholesterol transport
SD: standard deviation
T1D: type 1 diabetes
